# α-Synuclein structural features inhibit harmful polyunsaturated fatty acid oxidation, suggesting roles in neuroprotection

**DOI:** 10.1074/jbc.M116.765149

**Published:** 2017-02-23

**Authors:** Giorgia De Franceschi, Chiara Fecchio, Ronit Sharon, Anthony H. V. Schapira, Christos Proukakis, Vittorio Bellotti, Patrizia Polverino de Laureto

**Affiliations:** From the ‡ Department of Pharmaceutical Sciences, CRIBI, Biotechnology Centre, University of Padova, 35121 Padova, Italy,; the § Department of Biochemistry and Molecular Biology, IMRIC, The Hebrew University-Hadassah Medical School, 9112102 Jerusalem, Israel,; the ¶ Department of Clinical Neuroscience, Institute of Neurology, University College London, NW32PF London, United Kingdom,; the ‖ Wolfson Drug Discovery Unit, Centre for Amyloidosis and Acute Phase Proteins, Division of Medicine, University College London, London, United Kingdom, and; the ** Department of Molecular Medicine, Institute of Biochemistry, University of Pavia, 27100 Pavia, Italy

**Keywords:** α-synuclein (α-synuclein), lipid oxidation, mass spectrometry (MS), polyunsaturated fatty acid (PUFA), protein chemical modification

## Abstract

α-Synuclein (aS) is a protein abundant in presynaptic nerve terminals in Parkinson disease (PD) and is a major component of intracellular Lewy bodies, the pathological hallmark of neurodegenerative disorders such as PD. Accordingly, the relationships between aS structure, its interaction with lipids, and its involvement in neurodegeneration have attracted great interest. Previously, we reported on the interaction of aS with brain polyunsaturated fatty acids, in particular docosahexaenoic acid (DHA). aS acquires an α-helical secondary structure in the presence of DHA and, in turn, affects DHA structural and aggregative properties. Moreover, aS forms a covalent adduct with DHA. Here, we provide evidence that His-50 is the main site of this covalent modification. To better understand the role of His-50, we analyzed the effect of DHA on aS-derived species: a naturally occurring variant, H50Q; an oxidized aS in which all methionines are sulfoxides (aS4ox); a fully lysine-alkylated aS (acetyl-aS); and aS fibrils, testing their ability to be chemically modified by DHA. We show, by mass spectrometry and spectroscopic techniques, that H50Q and aS4ox are modified by DHA, whereas acetyl-aS is not. We correlated this modification with aS structural features, and we suggest a possible functional role of aS in sequestering the early peroxidation products of fatty acids, thereby reducing the level of highly reactive lipid species. Finally, we show that fibrillar aS loses almost 80% of its scavenging activity, thus lacking a potentially protective function. Our findings linking aS scavenging activity with brain lipid composition suggest a possible etiological mechanism in some neurodegenerative disorders.

## Introduction

**Figure 1.**
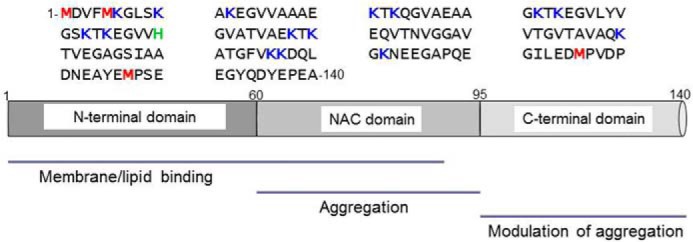
**Amino acid sequence of aS.** Schematic representation of the aS sequence highlighting methionine, lysine, and histidine residues in *red*, *blue*, and *green*, respectively (*top*). Scheme of the three major domains of the protein: the N-terminal region involved in lipid and membrane binding, the NAC domain (61–95) responsible for aggregation properties of the protein, and the acidic C-terminal region, able to transiently interact with the N-terminal and the NAC domains modulating the aggregation propensity of the protein (*bottom*).

α-Synuclein (aS)^3^ is a cytosolic protein abundant in presynaptic nerve terminals involved in the pathogenesis of Parkinson disease (PD). It is a major component of intracellular Lewy bodies, the pathological hallmarks of neurodegenerative disorders such as PD and Lewy body dementia (1). Several point mutations in aS gene (*SNCA*) and copy number gains have been found to lead to PD (2–5). Structurally, aS is classified as a natively unfolded protein, being disordered in solution (6). Its sequence is generally considered as consisting of three major domains (Fig. 1). The N-terminal, residues 1–60, contains four of the seven imperfect ∼11 amino acids repeats sharing similarity to lipid binding domains of apolipoproteins, and acquires helical conformation upon binding to lipids and membranes (7, 8). The central region, residues 61–95, non-Aβ component (NAC), has a critical role for nucleation of the aggregation process (9). The C-terminal, enriched in acidic residues and prolines, is highly hydrophilic, and able to establish transient interactions with the N-terminal or other proteins (10). aS physiological function is not yet fully defined, but several studies correlate the protein with membranes and lipids for its ability to remodel membranes (11), to influence the lipid packing (12), or to regulate the synaptic vesicle fusion (13, 14).

Previous reports documented the ability of aS to interact with brain polyunsaturated fatty acids (PUFAs), which are critical for neuronal membranes, fluidity, and permeability, serving as an energy reservoir, and taking part in intra- and extracellular signaling as second messengers (15). PUFAs mediate other functions such as generation of apoptotic signals, cellular proliferation, and translocation of lipid-modifying enzymes to cellular membranes (15). Docosahexaenoic (DHA, 22:6) and arachidonic (AA, 20:4) acids are the most abundant fatty acids in the brain. DHA is a *n-*3 fatty acid containing six double bonds, whereas AA is a *n*-6 fatty acid with four double bonds, therefore they are highly susceptible to oxidation. They can be oxidized enzymatically and non-enzymatically through a free radical-mediated mechanism (16–18). Each oxidation mechanism yields specific products, in particular hydroperoxides, hydroxide positional isomers, and free radicals that are highly reactive (19). The relative susceptibility of PUFAs to oxidation depends on the reaction milieu as well as their inherent structure. The biological role of PUFA peroxidation products has received a great deal of attention because when it was discovered that α,β-unsaturated aldehydes, the advanced lipid peroxidation end products, can react with sulfhydryl groups of proteins, forming stable adducts able to affect several metabolic processes (20).

Lipid peroxidation is involved in several human diseases such as atherosclerosis, cancer, diabetes, acute lung injury, as well as neurodegenerative disorders including PD (21), Huntington disease (22), and Alzheimer disease (23). Therefore, efforts have been devoted to understanding the mechanism of lipid peroxidation and preventing the deleterious effects of this process. Interestingly, it was shown that 4-hydroxy-2-nonenal (4HNE), a product of lipid peroxidation, forms protein adducts in Lewy bodies in neocortical and brain neurons (24). 4HNE was also found to alter dopamine transport, contributing to the loss of dopamine, which is a feature of PD (25). In this scenario, the correlation between aS and PUFAs has raised considerable interest.

The consequences of aS ability to interact with PUFAs are immediately ascertainable on its structure, because the protein undergoes conformational transition in the presence of fatty acids containing unsaturations (26, 27). It seems that the 1–70 region of aS is mainly involved in the molecular interaction with the fatty acid (27). We, in particular, showed that aS in the presence of DHA acquires α-helical secondary structure and forms, upon incubation, toxic oligomers able to affect membrane permeability (27, 28). The mutual effect between DHA and aS is strongly determined by the protein/lipid ratio. Furthermore, the protein undergoes chemical modifications, such as the formation of a covalent adduct with DHA, methionine (Met) residue oxidation, and carbonylation (29). The four Met residues (Met-1, Met-5, Met-116, and Met-127) are easily oxidized to sulfoxide and this phenomenon plays an important role in modulating aS membrane binding and aggregation (30). Methionine sulfoxide reductase reverses oxidized aS suggesting that the protein can contribute to the protection of membrane from oxidative damage (31). Moreover, it was observed that 4HNE formed under strong oxidative stress conditions can covalently bind aS, triggering the formation of β-sheet-rich oligomers (32). However, the relationship between aS structure, its binding to PUFAs, and the role of the chemical modification, is still not clear. It was proposed that aS may act physiologically as a catalytically regenerated scavenger of oxidants in healthy cells (33).

In the present work, we show by mass spectrometry analysis that the early radical products of DHA and AA autoxidation react with aS, producing a covalent modification on the protein. Histidine (His) at position 50 seems to be the major target of this reaction and the four Met are oxidized. To understand if the role of His would be specific in this sort of scavenger activity of free radicals, we analyze DHA in the presence of a pathogenic aS variant presenting the H50Q missense mutation (34–36). Then we evaluated a possible contribution of Met residues by using a completely oxidized aS and to understand the role of the 7 imperfect 11-residue repeats domain, all lysine (Lys) residues were acetylated. Finally, for a correlation with the aggregation state of aS, DHA was also analyzed in the presence of aS fibrils.

Our *in vitro* data suggest that aS might be able to exert a sequestering/scavenging activity *versus* DHA and AA early radical autoxidation products that is modulated by different factors. First, the mutual structural interaction between aS and PUFAs is an essential prerequisite for this activity, because it provides a stabilizing effect on the fatty acid physical state. Second, His and Lys collaborate in sequestering the early peroxidation products. In this way, the propagation of the oxidative process of fatty acids is hampered, and concentration of the highly reactive species is reduced.

## Results

### DHA and AA autoxidation process

**Figure 2.**
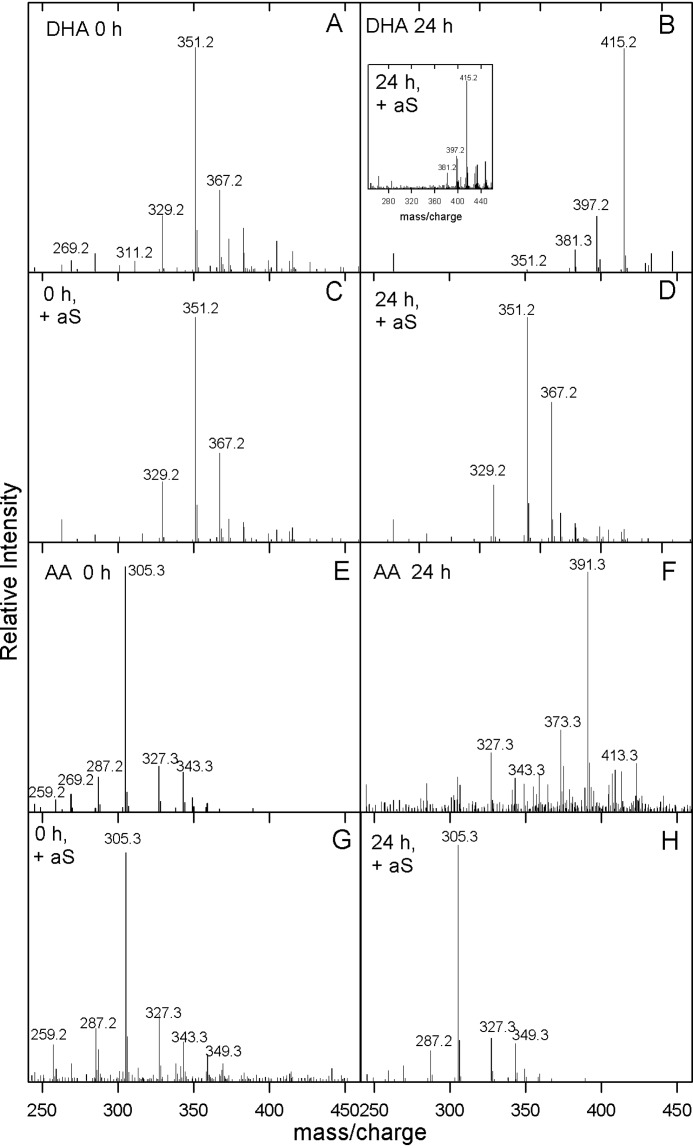
**Mass/charge spectra of DHA and AA.** Positive ESI-MS of DHA (*A–D*) and AA (*E* and *F*), recorded just after the preparation of the samples (*A* and *E*) and upon 24 h at 37 °C and 300 rpm (*B* and *F*). The same samples were analyzed in the presence of aS (P/lipid 1:50) at 0 h (*C* and *G*) and after 24 h of incubation (*D* and *H*). *B*, *inset*: as control, DHA upon 24 h at 37 °C in the presence of aS added immediately before the MS analysis. The spectra were obtained at +2.5 kV of capillary and a cone voltage of 35 V.

**Scheme 1.**
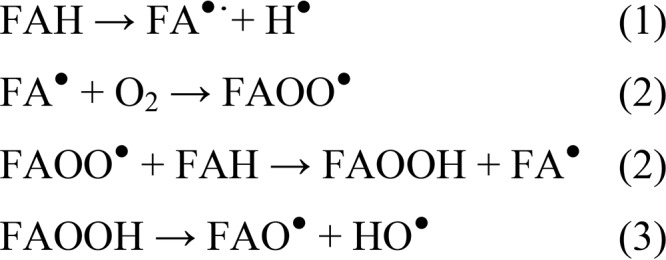
**Mechanism of non-enzymatic free-radical mediated oxidation of unsaturated lipids.** These chain reactions, described in terms of initiation (*1*), propagation (*2*), and termination (*3*), are also defined lipid autoxidation process (19). The initiation is induced by thermolysis, metal catalysis, or photolysis. Symbols here used: FAH, fatty acid; FA^•^, fatty acid radical; FAOO^•^, peroxyl radical, FAOOH, hydroperoxides; FAO^•^, alkoxy radical; HO^•^, hydroxy radical.

DHA and AA samples were incubated at pH 7 and 37 °C under shaking up to 24 h, in the absence and presence of aS (P/lipid molar ratio 1:50). Aliquots from DHA samples were analyzed by direct injection in electrospray ionization (ESI)-MS (Fig. 2). The ESI-MS spectrum of DHA just after its solubilization is reported in Fig. 2*A*. It presents a specific pattern of several signals (37): the most peculiar are those at mass/charge values of 329.2, 351.2, and 367.2 corresponding to a protonated molecular DHA (monoisotopic mass 328.2 Da), an adduct with sodium (328.2 Da + 22), and an oxidized form of this adduct (328.2 Da + 22 + 16). Moreover, other minor signals due to in-cone fragmentation (*m*/*z* 311.2, 269.2) are also visible. After 24 h of incubation (Fig. 2*B*), the signals at *m*/*z* 329.2, 351.2, and 367.2, characteristics of DHA, disappear and new species are evident in the spectrum at *m*/*z* values of 381.3, 397.2, and 415.2 corresponding to hydroperoxide derivatives of DHA, which generate during the propagation phase of the fatty acids autoxidation (Scheme 1). The species at *m*/*z* 415.2 is the most intense signal and it has been identified by MS-MS analysis, as a di-hydroperoxide derivative of DHA (Table 1). Then the fatty acid was incubated in the presence of aS recording the mass spectra up to 24 h (Fig. 2*D*). The 24-h spectrum is quite similar to that obtained at time 0 (Fig. 2, *A* and *C*) and the oxidized species do not form in the presence of the protein. As control, DHA upon 24 h at 37 °C in the presence of aS added immediately before the MS analysis, was analyzed to show that the higher molecular weight DHA species are still detectable.

**Table 1:** **DHA and AA products identified in their ESI spectra obtained in the absence and in the presence of aS (Fig. 2)**

MH^+^	Species	Description
329.2	*DHA (C_22_H_32_O_2_)*	Docosahexaenoic acid (monoisotopic mass 328.2)
351.2	*DHA* + Na	Sodium adduct of DHA
367.2	*DHA*OH + Na	Sodium adduct of hydroxy DHA
381.3	*DHA*OO^•^ + Na	Sodium adduct of peroxy radical DHA or
	*DHA*(O^•^)_2_ + Na	Sodium adduct of dialkoxy radical DHA
397.2	*DHA*(OO^•^)(O^•^) + Na	Sodium adduct of alkoxy-peroxy diradical DHA
415.2	*DHA*(OOH)_2_ + Na	Sodium adduct of dihydroperoxy DHA
305.3	*AA (C_20_H_32_O_2_)*	Arachidonic acid (monoisotopic mass 304.2)
327.3	*AA* + Na	Sodium adduct of AA
343.3	*AA*OH + Na	Sodium adduct of hydroxy AA
349.3	*AA* + 2Na	Di-sodium adduct of AA
373.3	*AA*(OOH)_2_ + Na − 18	Sodium adduct of dihydroperoxy AA dehydrated
391.3	*AA*(OOH)_2_ + Na	Sodium adduct of dihydroperoxy AA
413.3	*AA*(OOH)_2_ + 2Na	Di-sodium adduct of dihydroperoxy AA

The same experiment was conducted using AA as a fatty acid. In Fig. 2*E*, the *m*/*z* spectrum of AA is reported, where the signals corresponding to molecular AA, its sodium adduct, and its hydroxyl form are evident (*m*/*z* 305.3, 327.3, and 343.3). Also the products of partial degradation of AA (37) at *m*/*z* 259.2, 269.2, and 287.2 are visible. After 24 h of incubation, AA forms several species including the di-hydroperoxy derivative (*m*/*z* 391.3), a sign of autoxidation process (Fig. 2*F*). In Fig. 2, *G* and *H*, the same spectra are recorded in the presence of aS, showing that in this case the early autoxidation products do not form within 24 h of incubation with the protein. In Table 1, the species of DHA and AA identified by MS analysis are listed.

### Chemical modification of aS

The mass analysis of aS samples analyzed in the presence of DHA or AA are reported in Table 2. In particular, species containing oxidations (+16 Da or multiple of 16) and covalent adducts with DHA (+326 Da) or AA (+304 Da) are present. For the purpose of the present work, the samples were analyzed only up to 24 h of incubation, because aS aggregates and forms oligomers upon prolonged incubation in the presence of fatty acids, as previously observed, and other modifications can occur (29).

**Table 2:** **Molecular masses of aS after incubation in the presence of DHA or AA**

PUFA	Molecular mass (Da)		Protein species
	Found*^a^*	Calculated*^b^*	
	14459.9 (±0.1)	14460.1	aS
	14475.7 (±0.8)	14476.1	aS + 1ox
	14492.2 (±0.1)	14492.1	aS + 2ox
	14508.3 (±0.1)	14508.1	aS + 3ox
DHA	14802.2 (±0.1)	14802.1	aS + 1ox + 326
	14817.1 (±0.7)	14818.1	aS + 2ox + 326
	14835.3 (±0.5)	14834.1	aS + 3ox + 326
	14849.9 (±0.7)	14850.1	aS + 4ox + 326
	14865.2 (±0.9)	14866.1	aS + 3ox + 358
	14880.8 (±0.9)	14882.1	aS + 4ox + 358
AA	14747.2 (±1.4)	14746.1	aS + 286
	14765.1 (±0.8)	14764.1	aS + 304
	14781.2 (±0.9)	14780.1	aS + 1ox + 304
	14794.8 (±0.5)	14796.1	aS + 2ox + 304

*^a^* Experimental molecular masses determined by ESI-MS.

*^b^* Masses calculated from the amino acid sequence of aS.

**Figure 3.**
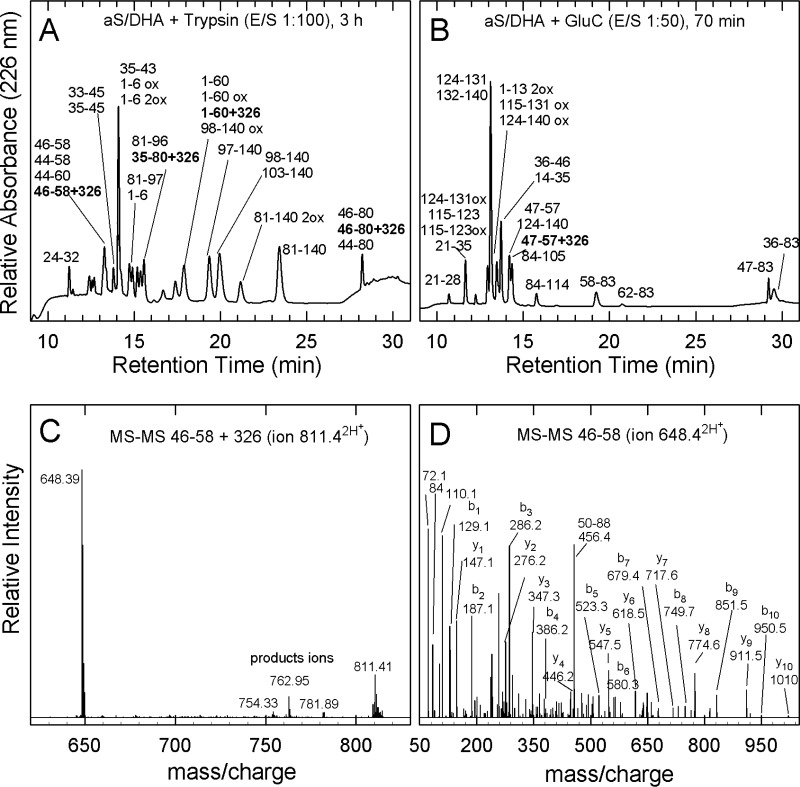
**Site of chemical modification in aS.** Identification of His-50 as the preferential site of chemical modification of aS in the presence of DHA. Fingerprinting analysis of chemically modified aS/DHA sample by trypsin (*A*) and endoprotease GluC (*B*). The analysis was conducted on a Vydac C18 column (4.6 × 250 mm; The Separations Group, Hesperia, CA), eluted with a gradient of water, 0.1% TFA *versus* acetonitrile, 0.085% TFA from 5 to 25% in 5 min, from 25 to 28% in 13 min, from 28 to 39% in 3 min, and from 39 to 45% in 21 min at a flow rate of 1 ml/min. The *numbers* close to the peaks refer to the main proteolytic fragments. The fragments containing the chemical modification are highlighted. MS-MS analysis of the double charged ion at 811.4 *m*/*z* corresponding to peptide-(46–58) containing the covalent modification (*C*). MS-MS analysis of the double charged ion at 648.4 *m*/*z* corresponding to the peptide-(46–58) (*D*).

To identify the site of modification in the aS sequence, fingerprinting analysis by trypsin and endoproteinase GluC was performed. These proteases selectively cleave the peptide bonds involving basic and glutamic acid residues, respectively (Fig. 3). A RP-HPLC fraction enriched of the chemically modified aS with DHA was digested by trypsin, using an enzyme to substrate ratio (E/S) of 1:100, or by GluC, using an E/S of 1:50. The proteolytic fragments were purified by RP-HPLC (Fig. 3, *A* and *B*) and identified by MS (Tables 3 and 4). In the tryptic mixture, fragments 1–6, 1–60, 81–140, and 98–140, containing Met residues are present also in oxidized form (+16 Da), whereas fragments corresponding to sequences 1–60, 35–80, 46–80, and 46–58 were found with a mass increase of 326 Da. The residues, susceptible of modification, present in all the detected modified peptides are His and Lys. His as a nucleophile is stronger than Lys, and it is consequently more prone to be modified. To confirm this hypothesis, MS-MS analysis of the modified fragments was performed. In Fig. 3*C*, the MS-MS spectrum of the peptide-(46–58) + 326, the shortest modified peptide, is reported. The analysis was conducted on its double charged ion at *m*/*z* 811.4. The spectrum does not show signals relative to the series of *y*- or *b*-ions, but products of partial degradation (*m*/*z* 754.33, 762.95, and 781.89) together with a strong ion at *m*/*z* 648.39 corresponding to the double charged ion of the peptide-(46–58) without the covalent adduct (Fig. 3*C*). The species at *m*/*z* 781.89 is particularly interesting, because it is produced for removal of the moiety CH_2_COOH (59 Da), partially confirming the identity of the modified peptide. From the other hand, it was not possible to obtain direct evidence of which specific amino acid has reacted with the fatty acid, because the energy used for the collision of the double charged ions produces ions corresponding to the peptide without modification (*m*/*z* 648.39). Indeed, the MS-MS spectrum of the ion at *m*/*z* 648.39 confirms the identity of the peptide (Fig. 3*D*). In the proteolytic mixture of modified aS with DHA by GluC, an increase of 326 Da was found on the mass of peptides corresponding to 36–61 and 29–57 after a 5-min reaction (data not shown) and to 47–57 after a 70-min reaction (Fig. 3*B*, Table 4). This last peptide, which does not contain Lys, provides direct evidence that His-50 is modified, even if, also in this case, a MS-MS spectrum was not obtained. Moreover, no fragments containing only Lys (and not His) were found modified.

**Table 3:** **Chemical characterization of fragments corresponding to the peaks of the chromatogram relative to the fragmentation of aS/DHA with trypsin shown in Fig. 3*A***

RT*^a^*	Molecular mass (Da)		Peptide species*^d^*
	Found*^b^*	Calculated*^c^*	
*min*			
11.2	830.43 (±0.01)	829.91	24–32
13.2	1294.99 (±0.05)	1294.68	46–58
	1524.16 (±0.01)	1523.74	44–58
	1620.8 (±0.1)	1621.46	**46–58 + 326**
	1754.37 (±0.02)	1754.02	44–60
13.8	1179.93 (±0.05)	1179.65	35–45
	1406.9 (±0.1)	1408.69	33–45
14.1	785.88 (±0.11)	785.98	1–6 + 1ox
	801.78 (±0.12)	801.98	1–6 + 2ox
	950.7 (±0.1)	950.09	35–43
14.7	2157.17 (±0.04)	2157.45	59–80
14.9	769.88 (±0.1)	769.98	1–6
	1607.16 (±0.01)	1606.84	81–97
15.1	2148.14 (±0.11)	2148.44	81–102
15.4	1928.41 (±0.05)	1928.17	61–80
15.6	1478.05 (±0.04)	1478.66	81–96
	4923.6 (±0.1)	4923.25	**35–80 + 326**
17.9	4847.06 (±0.38)	4846.03	98–140 + 1ox
	6150.38 (±0.1)	6149.18	1–60
	6167.3 (±0.5)	6165.18	1–60 + 1ox
	6472.55 (±0.75)	6475.18	**1–60 + 326**
19.3	4958.66 (±0.01)	4958.20	97–140
19.9	4288.74 (±0.34)	4288.43	103–140
	4830.5 (±0.1)	4830.03	98–140
21.2	6434.93 (±0.04)	6434.86	81–140 + 1ox
	6451.22 (±0.46)	6450.86	81–140 + 2ox
23.4	6418.65 (±0.13)	6418.86	81–140
28.2	3434.52 (±0.05)	3434.89	46–80
	3760.8 (±0.1)	3760.89	**46–80 + 326**
	3664.32 (±0.01)	3664.18	44–80

*^a^* Peptides are listed in order of retention times (RT).

*^b^* Experimental molecular masses determined by ESI-MS.

*^c^* Molecular masses calculated from aS amino acid sequence.

*^d^* The modified peptides are highlighted in bold.

**Table 4:** **Chemical characterization of peptide species corresponding to the peaks of the chromatogram relative to the fragmentation of aS/DHA with GluC, after 70-min incubation, shown in Fig. 3*B***

RT*^a^*	Molecular mass (Da)		Peptide species*^d^*
	Found*^b^*	Calculated*^c^*	
min			
4.5	703.21 (±0.01)	703.39	29–35
5.2	607.21 (±0.02)	607.25	136–140
10.8	859.52 (±0.01)	859.48	21–28
11.7	970.36 (±0.01)	970.36	124–131 + 1ox
	1029.61 (±0.01)	1030.39	115–123
	1045.72 (±0.02)	1046.39	115–123 + 1ox
	1544.85 (±0.01)	1544.85	21–35
12.5	615.46 (±0.01)	615.32	14–20
12.8	1998.61 (±0.02)	1998.74	115–131 + 2ox
12.9	1457.01 (±0.01)	1456.79	14–28
13.2	954.35 (±0.01)	954.36	124–131
	1069.62 (±0.01)	1070.42	132–140
13.3	1515.7 (±0.1)	1514.76	1–13 + 2ox
	1982.8 (±0.1)	1982.74	115–131 + 1ox
	2022.8 (±0.1)	2022.77	124–140 + 1ox
13.7	1179.71 (±0.02)	1179.65	36–46
	2142.2 (±0.1)	2142.16	14–35
13.8	912.32 (±0.03)	912.46	106–114
	1037.56 (±0.01)	1037.55	47–57
	2006.69 (±0.02)	2006.77	124–140
14.2	2112.08 (±0.01)	2112.07	1–20 + 2ox
14.3	1363.62 (±0.02)	1363.56	**47–57 + 326**
	2189.82 (±0.51)	2190.13	84–105
15.8	3083.89 (±0.03)	3084.57	84–114
19.3	2614.04 (±0.05)	2613.43	58–83
20.8	2127.16 (±0.01)	2127.15	62–83
29.3	3633.11 (±0.11)	3632.97	47–83
29.5	4795.66 (±0.02)	4794.61	36–83

*^a^* Peptides are listed in order of retention times (RT).

*^b^* Experimental molecular masses determined by ESI-MS.

*^c^* Molecular masses calculated from aS amino acid sequence.

*^d^* The modified peptides are highlighted in bold.

### Interaction of H50Q, aS-4ox, and acetyl-aS with DHA

**Figure 4.**
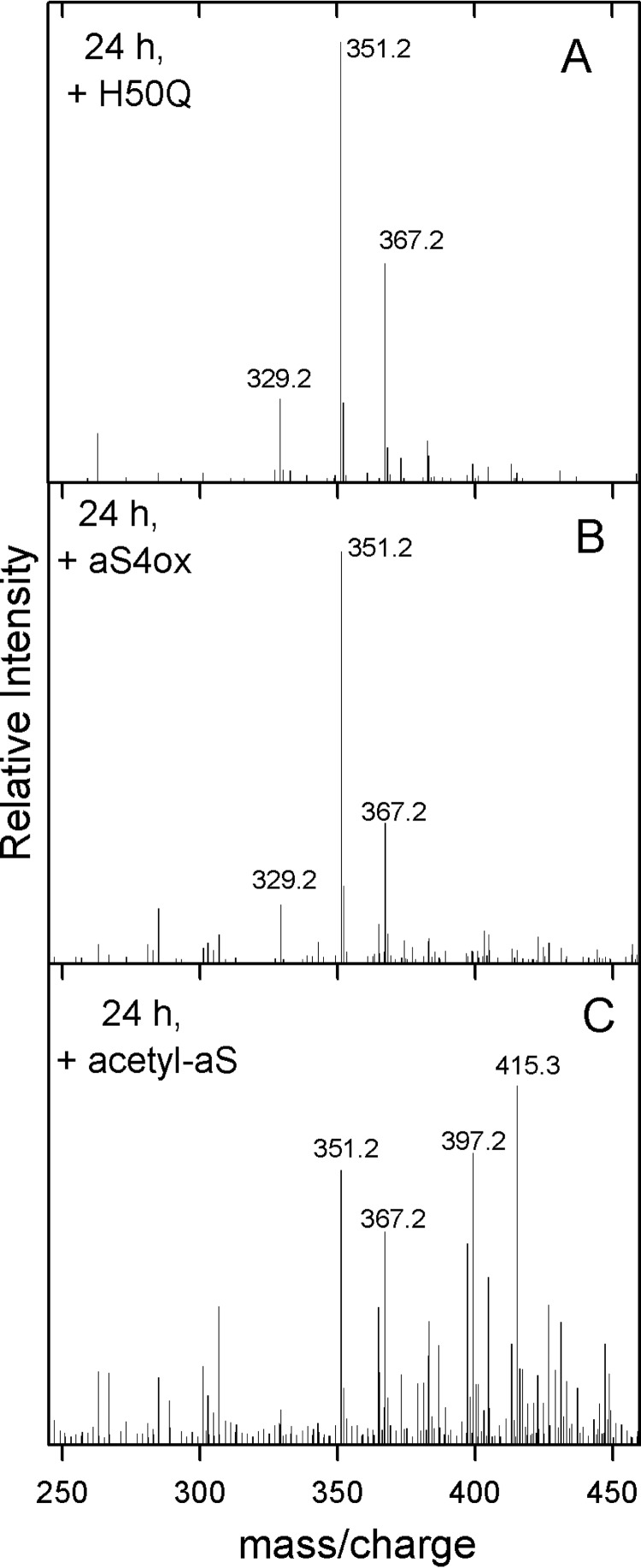
**Mass/charge spectra of DHA in the presence of H50Q, aS4ox, and acetyl-aS.** Positive ESI-MS spectra of DHA recorded upon incubation for 24 h at 37 °C and 300 rpm in the presence of H50Q (*A*), tetraoxidized aS (aS4ox, *B*), and alkylated aS (acetyl-aS, *C*). Proteins were added to realize a protein/lipid ratio of 1:50. Measurements are conducted as described in the legend to Fig. 2.

To understand the specific role of His as a site of modification of aS in the presence of fatty acids, the interaction with DHA was studied testing the variant in which the only His residue in position 50 is replaced by Gln. The role of Met was scrutinized by using aS with oxidized Met (tetraoxidized aS, aS4ox). Acetylation was used to abrogate the effect of the 15 Lys (acetyl-aS). In Fig. 4, the *m*/*z* spectra of DHA after 24 h of incubation in the presence of these three aS species are reported. They show that DHA early autoxidation products are not present if the fatty acid is incubated with H50Q (Fig. 4*A*) and aS4ox (Fig. 4*B*), which in turn are chemically modified by DHA and form covalent adducts (Table 5). On the contrary, in the presence of acetyl-aS, the autoxidation of DHA is not prevented (Fig. 4*C*) and, in the corresponding spectrum, the species at *m*/*z* 415.3, indicative of the formation of the di-hydroperoxide derivative of DHA, is visible and is the most intense signal.

**Table 5:** **Molecular masses of H50Q, aS4ox, and acetyl-aS in the presence of DHA**

Protein	Molecular mass (Da)		Protein species
	Found*^a^*	Calculated*^b^*	
H50Q	14453.2 (±0.1)	14451.1	H50Q
	14468.7 (±0.8)	14467.1	+ 1ox
	14485.2 (±0.1)	14483.1	+ 2ox
	14780.3 (±0.9)	14777.1	+ 326
	14795.3 (±0.1)	14793.1	+ 1ox + 326
	14811.1 (±0.7)	14809.1	+ 2ox + 326
	14826.7 (±0.5)	14825.1	+ 3ox + 326
	15100.5 (±1.8)	15103.1	+ 2 × 326
aS4ox	14525.1 (±1.1)	14524.1	aS4ox
	14542.1 (±0.7)	14540.1	+ 1ox
	14836.1 (±0.8)	14834.1	+ 310
	14852.8 (±0.9)	14850.1	+326
Acetyl-aS	14966.7 (±1.1)	14964.4	+12 acetyl
	15009.5 (±1.8)	15006.4	+13 acetyl
	15050.5 (±2.1)	15048.4	+14 acetyl
	15092.1 (±1.5)	15090.4	+15 acetyl
	15134.4 (±1.6)	15132.4	+16 acetyl

*^a^* Experimental molecular masses determined by ESI-MS.

*^b^* Molecular masses calculated from the amino acid sequence of aS species.

The mass values of aS species measured in the presence of DHA are reported in Table 5. Several species of acetyl-aS are observable in its mass spectrum, due to the fact that there are molecules in which not all the 15 Lys residues are acetylated and species also containing the N-terminal residue acetylated (+ 16 acetyl). There is no presence of adduct with DHA.

**Figure 5.**
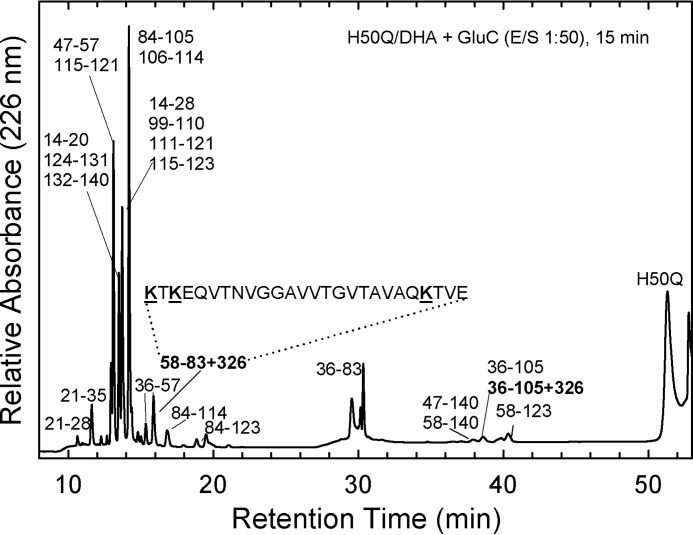
**Fingerprinting analysis of modified H50Q by GluC.** RP-HPLC analysis of the proteolytic mixture of modified H50Q with DHA. The analysis was conducted on a Vydac C18 column (4.6 × 250 mm; The Separations Group, Hesperia, CA), eluted with a gradient of water, 0.1% TFA *versus* acetonitrile, 0.085% TFA from 5 to 25% in 5 min, from 25 to 28% in 13 min, from 28 to 39% in 3 min, and from 39 to 45% in 21 min at a flow rate of 1 ml/min. The *numbers* close to the peaks refer to the main proteolytic fragments. The sequence of the peptide containing the chemical modification is reported.

In the case of H50Q more than one adduct was found, indicating that His is not the only one possible modification site. Indeed, after His, also Lys are residues highly prone to be modified (see “Discussion”). Thus, lacking the preferential site of modification (His-50), it is reasonable to expect more than one site to undergo modification with DHA, because potentially all of the 15 Lys can be modified with the same chemical propensity. To identify the site(s) of modification, a RP-HPLC fraction enriched of the chemically modified H50Q with DHA was digested by using endoproteinase GluC, using an enzyme to substrate ratio of 1:50. The mixture was analyzed by RP-HPLC (Fig. 5). As in the case of aS/DHA, peptides that contain Met are present also in oxidized form (Table 6). The smallest fragment that has been found modified by DHA corresponds to the sequence 58–83 (found mass 2939.7 Da, calculated 2613.98 + 326 Da). This species contains 3 Lys residues (in position 58, 60, and 80). Lys in position 60 is reported to be, after His-50, the target of the reaction with 4HNE (38). However, we detected more than one adduct with DHA, therefore different lysines can be involved.

**Table 6:** **Chemical characterization of fragments corresponding to the peaks of the chromatogram relative to the fragmentation of H50Q/DHA with GluC shown in Fig. 5**

RT*^a^*	Molecular mass (Da)		Peptide species*^d^*
	Found*^b^*	Calculated*^c^*	
*min*			
10.6	859.5 (±0.1)	859.48	21–28
11.6	1028.4 (±0.1)	1028.55	47–57
	1046.4 (±0.2)	1046.39	115–123 + 1ox
	1068.4 (±0.2)	1070.42	132–140
	970.4 (±0.5)	970.36	124–131 + 1ox
12.3	615.3 (±0.1)	615.32	14–20
12.9	1030.4 (±0.1)	1030.39	115–123
	1456.8 (±0.2)	1456.79	14–28
	1200.6 (±0.2)	1199.46	131–140
13.1	954.4 (±0.2)	954.36	124–131
	1070.4 (±0.1)	1070.42	132–140
13.5	1514.8 (±0.1)	1514.83	47–61
13.7	1179.6 (±0.1)	1179.65	36–46
	1028.6 (±0.1)	1028.55	47–57
14.1	912.5 (±0.1)	912.46	106–114
15.0	2191.3 (±0.5)	2190.19	36–57
15.3	2939.7 (±0.2)	2940.48	**58–83 + 326**
15.8	3086.3 (±0.1)	3086.41	84–114
16.8	1926.1 (±0.9)	1924.84	106–123
18.8	4101.1 (±0.8)	4099.46	84–123
19.4	2614.5 (±0.1)	2614.98	58–83
21.1	2128.3 (±0.5)	2127.15	62–83
29.5	5684.8 (±0.8)	5683.37	58–114
30.1	4789.6 (±0.9)	4788.48	36–83
30.3	3626.3 (±0.5)	3626.12	47–83
37.9	9733.9 (±1.1)	9729.97	47–140 + 2ox
38.5	8676.1 (±0.5)	8676.56	47–131 + 2ox
39.8	6964.3 (±1.5)	6961.89	36–105
	7290.3 (±1.1)	7287.89	**36–105 + 326**
40.3	6696.6 (±1.1)	6696.42	58–123

*^a^* Peptides are listed in order of retention times (RT).

*^b^* Experimental molecular masses determined by ESI-MS.

*^c^* Molecular masses calculated from aS amino acid sequence.

*^d^* The modified peptides are highlighted in bold.

### Structural interaction with the fatty acids

**Figure 6.**
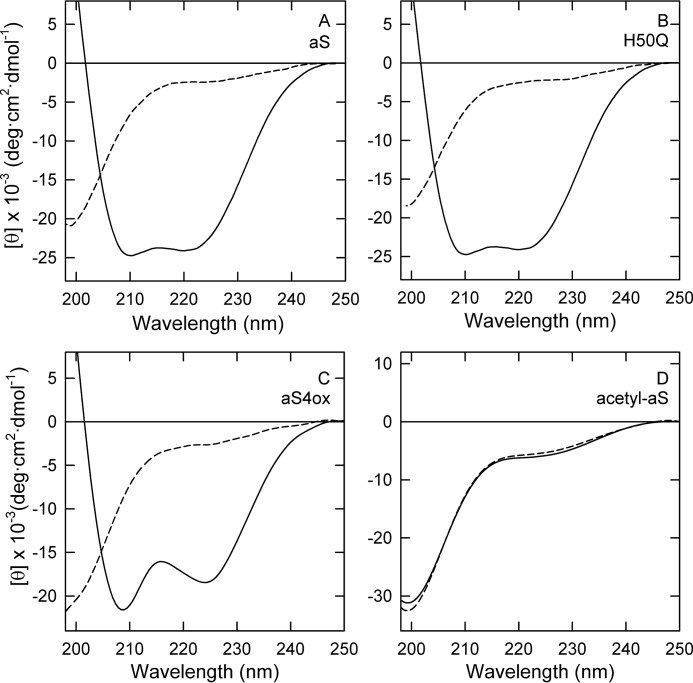
**Structural analysis by CD.** Far UV CD spectra of aS (*A*), H50Q (*B*), aS4ox (*C*), and acetyl-aS (*D*), in the absence (*dashed line*) and presence (*solid line*) of DHA (P/DHA, 1:50). The spectra were recorded in PBS buffer, pH 7.4, using a 1-mm path length quartz cell and a protein concentration of 5–7 μm.

Far UV-CD measurements were performed (Fig. 6), to correlate the ability of the protein to be modified with their structural features deriving from the interaction with DHA. H50Q and aS4ox maintain the property observed in aS to undergo structural transition acquiring the α-helical secondary structure in the presence of the polyunsaturated fatty acid (Fig. 6, *A–C*). Indeed, the characteristic signals at 208 and 222 nm in the far UV-CD spectra of the protein species in the presence of DHA are visible. The acetylation of lysine residues and the N-terminal amino group causes the loss of this ability and acetyl-aS remains unfolded in the presence of the fatty acid (Fig. 6*D*). Interestingly, this correlates with the loss of scavenger activity observed in Fig. 4*C*, whereas all the other interacting species maintain their ability to slow down DHA oxidation.

### DHA and aS fibrils

**Figure 7.**
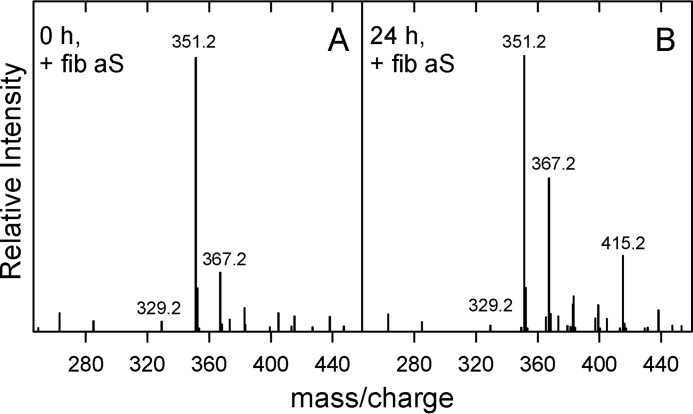
**DHA and aS fibrils.** Positive ESI-MS spectra of DHA recorded after 0 (*A*) and 24 h (*B*) at 37 °C and 500 rpm in the presence of preformed fibrils of aS. Fibrils are added to realize a protein to fatty acid ratio of 1:50.

MS spectra of DHA were recorded in the presence of preformed WT aS fibrils (Fig. 7*A*). After 24 h of incubation (Fig. 7*B*), the mass/charge signals at 329.2, 351.2, and 367.2 are still evident, but the presence of the signal at *m*/*z* 415.2 shows that DHA undergoes partial oxidation. aS fibrils were further oxidized as observed by MS analysis (not shown).

## Discussion

The relationship between aS and brain PUFAs is a debated, but important question. It provides a link for the onset of PD (39). In the presence of DHA and AA, aS undergoes deep structural changes, from the acquisition of stable secondary α-helical structure to oligomerization or accelerated aggregation, upon incubation, as a function of the ratio between the protein and the fatty acid (27–29). Other consequences of this interaction are the chemical modifications of the side chain of certain residues, such as the oxidation of Met, the carbonylation, and the formation of covalent adducts (28). In this work, we show that His-50 is primarily modified in aS in the presence of PUFAs, for the formation of a covalent adduct. This raises the question if this effect would be specific and could be ascribable to a sequestering ability of aS to capture free radicals, thus playing a neuroprotective role in neurons in response to oxidative stress and eventually preventing oxidation of unsaturated fatty acids.

The process of unsaturated fatty acids peroxidation is a free-radical chain reaction that consists of three partially overlapping phases of radical reactions: initiation, propagation, and termination (17) (Scheme 1). Initiation takes place by abstraction of a hydrogen radical from the PUFA (FAH). During the propagation, the resulting lipid free radical (FA^•^) reacts with molecular oxygen to generate a peroxyl radical (FAOO^•^), which is the dominant radical present in the chain. It can react with another FAH to form hydroperoxides (FAOOH) and FA^•^, the early products of autoxidation. Successively, the hydroperoxides may decompose in alkoxy (FAO^•^) and hydro (HO^•^) derivatives. *In vivo*, the process is fostered by the high concentration of the oxygen (at millimolar levels) in the lipid bilayer (40). *In vitro*, the presence of trace metals or sources of energy (*i.e.* light or heat) can initiate the process (19). PUFAs easily undergo autoxidation because of the intrinsic structure of the radical intermediates, where electrons are highly delocalized. Normally, such radicals can interact with proteins giving rise to a wide range of compounds able to affect many physiological events (41). Here, we show that the propagation chain reaction is hampered in the presence of aS, and mutually, the protein results chemically modified with the formation of covalent adducts.

Fingerprinting analysis of the modified species and relative reactivities toward oxidative modifications suggested that the residue that is likely modified in aS is His-50. These data agree with the specific reactivity of lipid peroxidation products, such as allylic radicals, which involve preferentially in the order cysteine (absent in aS), histidine and lysine residues (42), and 4HNE, which, in aS, involves specifically His-50 (43). The imidazole moiety is nucleophilic and can react with one of the early products of autoxidation of DHA or AA. The propagation step in the PUFAs oxidation is hampered and the consequent breakdown into lipid-derived aldehydes and ketones is avoided, indeed, under our experimental conditions, the reaction terminates with the sequestration of the DHA or AA early reactive species by aS and the formation of a 4-hydroxy-2(E)-nonenal moiety was not observed.

Interestingly, Zhu *et al.* (44) proposed for aS an antioxidant activity of protection of lipids in neuronal membranes. They suggested a mechanism mediated by the four Met residues. This fact is corroborated by the finding that oxidized Met(s) can be repaired *in vivo* by intracellular methionine sulfoxide reductase (45). Our data show that the scavenging activity of DHA radicals is not exclusively correlated to Met residues, because aS4ox is still able to sequester these radicals. In view of the fact that His-50 is the only His residue in aS and, chemically, the preferential modification site by lipid radicals (42), the importance of this residue in the scavenger activity was investigated by using a natural aS variant containing a Gln instead of His in position 50 (H50Q) (34–36). We showed that, despite the lack of His-50, this variant is able to protect DHA. The behavior of H50Q in the presence of DHA is very similar to that of WT aS, and the protein acquires α-helical secondary structure upon incubation with DHA. It was recently found that the H50Q mutation does not affect aS subcellular localization, the structure of free or membrane-bound aS monomer nor its capacity to be phosphorylated *in vitro* (46). Moreover, the substitution of His-50 with Gln accelerates the conversion of aS into β-sheet-rich oligomers (35) and increases aS secretion and extracellular toxicity (46). If incubated with DHA, H50Q is modified and a mass increase of 326 Da (or a multiple of it) is still detectable, indicating the formation of covalent adduct(s) with the fatty acid. Lacking His, the modifications can reasonably occur at the level of the lysine residues, which, as nucleophiles, are vulnerable to modification by lipid peroxidation-derived electrophiles (42). A possible candidate is Lys in position 60 that was reported modified in the presence of 4HNE (38).

Our data show also that the structural interaction is an important requisite for aS scavenger activity. The analysis of the structural properties of aS4ox by CD studies has shown that the presence of methionine sulfoxide does not alter the ability of aS to acquire α-helical secondary structure upon interaction with DHA, as already observed with other lipids (30). On the contrary, the extensive acetylation does not allow any interaction with the fatty acid and acetyl-aS does not sequester DHA radicals. This behavior suggests an important role for Lys residues in the repeats of aS.

A further consideration can be done on the ability of aS fibrils to partially protect DHA. The fold of aS fibrils comprises a central core of cross-β structure (residues 35–94 according to Vilar *et al.* (47), and residues 38–96 according to Comellas *et al.* (48) flanked by flexible regions. In both models, the N-terminal domain containing the first and second repeats is not engaged in the fibril structure. Zarbiv *et al.* (49) demonstrated that lysines belonging to these repeats are essential for lipid interaction. Therefore, aS fibrils could still interact with DHA. The reduced activity could be due to the fact that not the entire N-terminal region, but likely only the first 25 residues, not engaged in the structure of fibril, are suitable for lipid interaction. This is consistent with the observation that the first 25 residues have the highest membrane affinity (50), with amino acids 6–25 likely firmly anchored to the membrane (51). Comparing the spectra of DHA obtained in the presence of aS as a monomer and aS as fibril, just evaluating the height of the signals at *m*/*z* 415 in a ratio with those due to not oxidized DHA, roughly fibrils lose 80% of the scavenging activity of aS. It is not unexpected that aS, accumulating as fibrils in Lewy bodies, does not maintain the scavenging activity. *In vivo*, fibrils formation could be further detrimental, because it is known that monomeric aS decreases in PD (52), probably captured by the fibrillar aggregates.

In our previous works, we have shown that aS and its N-terminal fragments (such as 1–52) reduce the aggregative concentration of DHA and cause a resizing of the lipid oil droplets with formation of a microemulsion (27, 53). In aqueous buffer, DHA forms an emulsion and shows a strong tendency to oxidize, generating monohydroxides and dihydroperoxides derivatives. In a bulk phase or in an inorganic solvent, this tendency is partially reduced (54). According to Miyashita (54), we can hypothesize that aS protects the fatty acid from oxidation acting also as an emulsifier. Indeed, when aS is not able to interact with lipids or even to resize the fatty acid droplets, no oxidant scavenger effect is observable. This is evident with acetyl-aS that lost the ability to interact with DHA (Fig. 6) and to resize DHA droplets (data not shown). However, aS does not behave as an inert emulsifier, but it undergoes oxidative modifications. Upon interaction with DHA, aS acquires a conformation that promotes its oxidation serving as terminal of the lipid oxidation process.

Several studies reported that aS is involved in the maintenance of the correct levels of PUFAs (55). It was also proposed that aS may play a protective role of neurons exposed to oxidative stress preventing oxidation of PUFAs (33). Indeed, lipid peroxidation increases with aging and is linked to PD (21). In neurons exposed to oxidative stress or obtained from patients affected by PD, the levels of PUFAs in a non-esterified form were found higher than normal (15). Interestingly, it was also reported that in cultured cells a PUFA treatment induces the appearance of toxic aS oligomers (56). Soluble oligomers are also triggered by oxidation of aS Met residues (57). *In vivo*, methionine sulfoxides are repaired by intracellular reductase. Very recently, it was observed that when the two C-terminal Met are not efficiently repaired by endogenous cellular enzymes, they remain oxidized. This results in the accumulation of oxidized aS, with high tendency to form oligomers, and that shows impaired phosphorylation (58). The cellular impact of this depends on the eventual toxicity of the oligomeric species. If toxic oligomers accumulate in cells, the formation of oxidized or partially oxidized aS becomes a significant contributing factor to PD. The protective role of aS toward PUFAs and the tendency of the protein to aggregate in their presence are apparently conflicting. Therefore, it is plausible to speculate that the protein/PUFAs ratio is a discriminant factor determining the effect of the interaction between aS and PUFAs. Under physiological conditions, aS can participate actively to the control of oxidative homeostasis of the intracellular environment, protecting the free fatty acids from oxidation and maintaining their correct level. Environmental factors can affect this equilibrium, by either increasing oxidative stress or changing DHA concentration. PD has been previously linked to oxidative stress, which can increase lipid oxidative species to an extent that does not allow the normal biological turnover of aS, leading to the accumulation of modified protein and to increased propensity to its oligomerization. Moreover, also the DHA concentration has been reported to increase in neuronal membranes of PD patients and also in general aging (55). Higher concentrations of DHA prevents aS amyloid aggregation, probably by the α-helix structure acquisition, but the formation of stable toxic oligomers is observed (27–29, 55). Because oligomers might represent the most toxic species, the increased DHA concentration in the cell could accelerate toxicity rather than having a protective role. Otherwise, we found that fibrils, in comparison to soluble aS, have a reduced scavenging activity. Determination of the real intracellular proportion between soluble and fibrillar aS will allow in the future to properly frame these findings within the complexity of the pathophysiology of the disease.

In conclusion, aS sequence is tailored to efficiently exert an oxidant scavenger activity, accordingly with recent hypothesis (33). The role of Met has been extensively described (30, 45). Lysine residues could play a significant role. Their distribution makes the aS interaction with PUFAs and other lipids effective. This deep interaction has specific consequences on the physical state of PUFAs, which are emulsified and resized as well as on the reactive species that are sequestered. Lysine and histidine residues can mediate the capturing action for its intrinsic chemical reactivity, hampering the propagation of the PUFAs oxidation process. This proposed scavenging mechanism could not be assumed as specific of aS. Its biological significance resides in the recognized role of the protein in PD, and specifically in the reported evidences of its interaction with PUFA and its involvement in their metabolism (11–16). aS is mainly a presynaptic protein, and it has been reported to be able to bind plasma membranes, lipid rafts, inner nuclear membrane, and mitochondrial membranes. These interactions are considered critical for the physiological function of aS (10). DHA is an essential PUFA, fundamental for the functions and maintenance of the nervous system, and deficits in DHA are associated with cognitive decline during aging and in neurodegenerative disease (59). aS has been repeatedly reported to interact with PUFA: endogenous cytosolic levels of aS can buffer arachidonic acid affecting SNARE assembly on cellular membrane (60). Elevated PUFA levels have been detected in PD or Lewy body dementia-affected human brains, whereas the levels of certain PUFAs were decreased in the brains of mice genetically deleted of aS (55). This mechanism of scavenging activity could provide a link between aS, altered lipid composition in neurodegenerative disorders, and PD development.

## Experimental procedures

### Protein expression and purification

Expression and purification of recombinant aS and aS variant were obtained as previously described (29, 35). Protein concentrations were determined by absorption measurements at 280 nm using a double-beam Lambda-20 spectrophotometer from PerkinElmer Life Sciences. The extinction coefficient for aS and H50Q at 280 nm was 5960 m^−1^ cm^−1^ (61). DHA and AA were purchased from Sigma. All other chemicals were of analytical reagent grade and were obtained from Sigma or Fluka (Buchs, Switzerland). Aliquots of DHA and AA were stored at a concentration of 76 mm in 100% ethanol at −80 °C under a helium atmosphere to prevent oxidation. Fibrils were obtained after 2 weeks of incubation of aS in PBS buffer, pH 7.4, using a protein concentration of 70 μm, at 37 °C under shaking (500 rpm). To isolate fibrils from monomers, the protein sample was ultracentrifuged at 380,000 × *g* for 1 h at 4 °C.

### Chemical modification of lysine and methionine residues in aS

The oxidation of the four Met residues was obtained using a method described in Uversky *et al.* (62). Briefly the protein was suspended in 20 mm phosphate buffer, 100 mm NaCl, pH 7, at a protein concentration of 3 mg/ml and H_2_O_2_ was added to a final concentration of 4% (v/v). The reaction was kept at room temperature for 20 min. The acetylation of Lys was performed according to Means and Feeney (63). aS was suspended in a 1:1 solution of water and saturated sodium acetate (protein final concentration 7.5 mg/ml), cooled in an ice-bath and treated with a total amount of acetic anhydride approximately equivalent to the weight of the protein, gradually added in 1 h. The solution was maintained at pH 8.5. The reagents were removed by using a centrifugal filter device (Amicon Ultra 10K, Millipore). The reaction mixtures were purified by RP-HPLC and the chemical modifications were verified by ESI-MS.

### Mass spectrometry

DHA and AA samples were analyzed by ESI-mass spectrometry (direct injection or upon purification in RP-HPLC) in the absence or in the presence of different protein species ([protein], 50 μm): aS, the variant H50Q, tetraoxidized aS (at the level of Met residues), and alkylated aS (at the level of Lys residues). The reaction was conducted in 20 mm phosphate buffer, pH 7.0, by using a molar protein/lipid ratio of 1:50, in which all aS molecules were bound to DHA and in α-helical conformation (27). DHA under this condition forms oil droplets, as previously reported (27). Aliquots of the solution after preparation and 24 h of incubation (37 °C, 300 rpm) were diluted 10 times in 50% H_2_O, 49.9% acetonitrile, 0.1% HCOOH, and analyzed by an ESI-mass spectrometer with a Q-TOF analyzer (Micro) from Waters (Manchester, UK). The measurements were conducted at a capillary voltage of 2.7 kV and a cone voltage of 35 V. The molecular masses of protein samples were estimated using the Mass-Lynx software 4.1 (Micromass).

### CD spectroscopy

Circular dichroism (CD) spectra in the far UV were recorded on a Jasco J-710 spectropolarimeter (Tokyo, Japan), using a 1-mm path length quartz cell and a protein concentration of 5–7 μm. The mean residue ellipticity [θ]_MRW_ (deg cm^2^ dmol^−1^) was calculated from the formula [θ]_MRW_ = (θ_obs_/10) (MRW/*lc*), where θ_obs_ is the observed ellipticity in deg, MRW is the mean residue molecular weight of the protein, *l* the optical path length in cm, and *c* the protein concentration in g/ml. The spectra were recorded in PBS buffer, pH 7.4.

### Fingerprinting analysis

Proteolysis with trypsin was conducted using an E/S ratio of 1:100 (by weight), in PBS, pH 7.4, and the reaction was quenched by acidification with TFA in water (4%, v/v). Proteolysis with endoproteinase GluC was performed at E/S ratio of 1:50 (by weight). RP-HPLC of proteolytic mixture was conducted on a Vydac C18 column (4.6 × 250 mm; The Separations Group, Hesperia, CA), eluted with a gradient of water, 0.1% TFA *versus* acetonitrile, 0.085% TFA from 5 to 25% in 5 min, from 25 to 28% in 13 min, from 28 to 39% in 3 min, and from 39 to 45% in 21 min at a flow rate of 1 ml/min. The peptides were identified by mass spectrometry. MS-MS analysis was conducted on the selected ions using collision energies from 15 to 30 V.

## Author contributions

P. P. D. L. conceived and supervised the project; G. D. F. and C. F. designed and performed the experiments; G. D. F., C. F., C. P., V. B., and P. P. D. L. analyzed the data; G. D. F., C. F., and P. P. D. L. wrote the paper; V. B. critically revised the paper; and R. S. and A. H. V. S. supervised parts of the project. All authors analyzed the results and approved the final version of the manuscript.

## Footnotes

3 The abbreviations used are:

aS: α-synuclein
PD: Parkinson's disease
NAC: non-Aβ component
PUFA: polyunsaturated fatty acid
DHA: Docosahexaenoic acid
AA: arachidonic acid
4HNE: 4-hydroxy-2-nonenal
ESI: electrospray ionization.

## References

[1] Spillantini M. G., Crowther R. A., Jakes R., Hasegawa M., and Goedert M. (1998) α-Synuclein in filamentous inclusions of Lewy bodies from Parkinson's disease and dementia with Lewy bodies. Proc. Natl. Acad. Sci. U.S.A. 95, 6469–6473 9600990 10.1073/pnas.95.11.6469 PMC27806

[2] Polymeropoulos M. H., Lavedan C., Leroy E., Ide S. E., Dehejia A., Dutra A., Pike B., Root H., Rubenstein J., Boyer R., Stenroos E. S., Chandrasekharappa S., Athanassiadou A., Papapetropoulos T., Johnson W. G., et al (1997) Mutation in the α-synuclein gene identified in families with Parkinson's disease. Science 276, 2045–2047 9197268 10.1126/science.276.5321.2045

[3] Krüger R., Kuhn W., Müller T., Woitalla D., Graeber M., Kösel S., Przuntek H., Epplen J. T., Schöls L., and Riess O. (1998) Ala30Pro mutation in the gene encoding alpha-synuclein in Parkinson's disease. Nat. Genet. 18, 106–108 9462735 10.1038/ng0298-106

[4] Zarranz J. J., Alegre J., Gómez-Esteban J. C., Lezcano E., Ros R., Ampuero I., Vidal L., Hoenicka J., Rodriguez O., Atarés B., Llorens V., Gomez Tortosa E., del Ser T., Muñoz D. G., and de Yebenes J. G. (2004) The new mutation, E46K, of α-synuclein causes Parkinson and Lewy body dementia. Ann. Neurol. 55, 164–173 14755719 10.1002/ana.10795

[5] Singleton A. B., Farrer M., Johnson J., Singleton A., Hague S., Kachergus J., Hulihan M., Peuralinna T., Dutra A., Nussbaum R., Lincoln S., Crawley A., Hanson M., Maraganore D., Adler D. C., et al (2003) α-Synuclein locus triplication causes Parkinson's disease. Science 302, 841 14593171 10.1126/science.1090278

[6] Weinreb P. H., Zhen W., Poon A. W., Conway K. A., and Lansbury P. T. Jr. (1996) NACP, a protein implicated in Alzheimer's disease and learning, is natively unfolded. Biochemistry 35, 13709–13715 8901511 10.1021/bi961799n

[7] Davidson W. S., Jonas A., Clayton D. F., and George J. M. (1998) Stabilization of α-synuclein secondary structure upon binding to synthetic membranes. J. Biol. Chem. 273, 9443–9449 9545270 10.1074/jbc.273.16.9443

[8] Jo E., McLaurin J., Yip C. M., St George-Hyslop P., and Fraser P. E. (2000) α-Synuclein membrane interactions and lipid specificity. J. Biol. Chem. 275, 34328–34334 10915790 10.1074/jbc.M004345200

[9] Uéda K., Fukushima H., Masliah E., Xia Y., Iwai A., Yoshimoto M., Otero D. A., Kondo J., Ihara Y., and Saitoh T. (1993) Molecular cloning of cDNA encoding an unrecognized component of amyloid in Alzheimer disease. Proc. Natl. Acad. Sci. U.S.A. 90, 11282–11286 8248242 10.1073/pnas.90.23.11282 PMC47966

[10] Snead D., and Eliezer D. (2014) α-Synuclein function and dysfunction on cellular membrane. Exp. Neurobiol. 23, 292–313 25548530 10.5607/en.2014.23.4.292 PMC4276801

[11] Jiang Z., de Messieres M., and Lee J. C. (2013) Membrane remodeling by α-synuclein and effects on amyloid formation. J. Am. Chem. Soc. 135, 15970–15973 24099487 10.1021/ja405993r PMC3859146

[12] Ouberai M. M., Wang J., Swann M. J., Galvagnion C., Guilliams T., Dobson C. M., and Welland M. E. (2013) α-Synuclein senses lipid packing defects and induces lateral expansion of lipids leading to membrane remodeling. J. Biol. Chem. 288, 20883–20895 23740253 10.1074/jbc.M113.478297 PMC3774359

[13] Burré J., Sharma M., Tsetsenis T., Buchman V., Etherton M. R., and Südhof T. C. (2010) α-Synuclein promotes SNARE-complex assembly *in vivo* and *in vitro*. Science 329, 1663–1667 20798282 10.1126/science.1195227 PMC3235365

[14] Burré J., Sharma M., and Südhof T. C. (2014) α-Synuclein assembles into higher-order multimers upon membrane binding to promote SNARE complex formation. Proc. Natl. Acad. Sci. U.S.A. 111, E4274–4283 25246573 10.1073/pnas.1416598111 PMC4210039

[15] Ruipérez V., Darios F., and Davletov B. (2010) α-Synuclein, lipids and Parkinson's disease. Prog. Lipid Res. 49, 420–428 20580911 10.1016/j.plipres.2010.05.004

[16] Niki E., Yoshida Y., Saito Y., and Noguchi N. (2005) Lipid peroxidation: mechanisms, inhibition, and biological effects. Biochem. Biophys. Res. Commun. 338, 668–676 16126168 10.1016/j.bbrc.2005.08.072

[17] Yin H., Xu L., and Porter N. A. (2011) Free radical lipid peroxidation: mechanisms and analysis. Chem. Rev. 111, 5944–5972 21861450 10.1021/cr200084z

[18] Derogis P. B., Freitas F. P., Marques A. S., Cunha D., Appolinário P. P., de Paula F., Lourenço T. C., Murgu M., Di Mascio P., Medeiros M. H., and Miyamoto S. (2013) The development of a specific and sensitive LC-MS-based method for the detection and quantification of hydroperoxy- and hydroxydocosahexaenoic acids as a tool for lipidomic analysis. PLoS ONE 8, e77561 24204871 10.1371/journal.pone.0077561 PMC3812029

[19] Frankel E. N. (1984) Chemistry of free radical and singlet oxidation of lipids. Prog. Lipid Res. 23, 197–221 6100997 10.1016/0163-7827(84)90011-0

[20] Reed T. T. (2011) Lipid peroxidationand neurodegenerative disease. Free Radic. Biol. Med. 51, 1302–1319 21782935 10.1016/j.freeradbiomed.2011.06.027

[21] Tsang A. H., and Chung K. K. (2009) Oxidative and nitrosative stress in Parkinson's disease. Biochim. Biophys. Acta 1792, 643–650 19162179 10.1016/j.bbadis.2008.12.006

[22] Pérez-De La Cruz V., Elinos-Calderón D., Robledo-Arratia Y., Medina-Campos O. N., Pedraza-Chaverrí J., Ali S. F., and Santamaría A. (2009) Targeting oxidative/nitrergic stress ameliorates motor impairment, and attenuates synaptic mitochondrial dysfunction and lipid peroxidation in two models of Huntington's disease. Behav. Brain Res. 199, 210–217 19100293 10.1016/j.bbr.2008.11.037

[23] Galasko D., and Montine T. J. (2010) Biomarkers of oxidative damage and inflammation in Alzheimer's disease. Biomark. Med. 4, 27–36 20383271 10.2217/bmm.09.89 PMC2850111

[24] Dalfó E., Portero-Otín M., Ayala V., Martínez A., Pamplona R., and Ferrer I. (2005) Evidence of oxidative stress in the neocortex in incidental Lewy body disease. J. Neuropathol. Exp. Neurol. 64, 816–830 16141792 10.1097/01.jnen.0000179050.54522.5a

[25] Morel P., Tallineau C., Pontcharraud R., Piriou A., and Huguet F. (1998) Effects of 4-hydroxynonenal, a lipid peroxidation product, on dopamine transport and Na+/K+ ATPase in rat striatal synaptosomes. Neurochem. Int. 33, 531–540 10098723 10.1016/s0197-0186(98)00062-x

[26] Broersen K., van den Brink D., Fraser G., Goedert M., and Davletov B. (2006) α-Synuclein adopts an α-helical conformation in the presence of polyunsaturated fatty acids to hinder micelle formation. Biochemistry 45, 15610–15616 17176082 10.1021/bi061743l

[27] De Franceschi G., Frare E., Bubacco L., Mammi S., Fontana A., and de Laureto P. P. (2009) Molecular insights into the interaction between α-synuclein and docosahexaenoic acid. J. Mol. Biol. 394, 94–107 19747490 10.1016/j.jmb.2009.09.008

[28] Fecchio C., De Franceschi G., Relini A., Greggio E., Dalla Serra M., Bubacco L., and Polverino de Laureto P. (2013) α-Synuclein oligomers induced by docosahexaenoic acid affect membrane integrity. PLoS ONE 8, e82732 24312431 10.1371/journal.pone.0082732 PMC3843715

[29] De Franceschi G., Frare E., Pivato M., Relini A., Penco A., Greggio E., Bubacco L., Fontana A., and de Laureto P. P. (2011) Structural and morphological characterization of aggregated species of α-synuclein induced by docosahexaenoic acid. J. Biol. Chem. 286, 22262–22274 21527634 10.1074/jbc.M110.202937 PMC3121372

[30] Glaser C. B., Yamin G., Uversky V. N., and Fink A. L. (2005) Methionine oxidation, α-synuclein and Parkinson's disease. Biochim. Biophys. Acta 1703, 157–169 15680224 10.1016/j.bbapap.2004.10.008

[31] Maltsev A. S., Chen J., Levine R. L., and Bax A. (2013) Site-specific interaction between α-synuclein and membranes probed by NMR-observed methionine oxidation rates. J. Am. Chem. Soc. 135, 2943–2946 23398174 10.1021/ja312415q PMC3585462

[32] Bae E. J., Ho D. H., Park E., Jung J. W., Cho K., Hong J. H., Lee H. J., Kim K. P., and Lee S. J. (2013) Lipid peroxidation product 4-hydroxy-2-nonenal promotes seeding-capable oligomer formation and cell-to-cell transfer of α-synuclein. Antioxid. Redox Signal. 18, 770–783 22867050 10.1089/ars.2011.4429 PMC3555112

[33] Schildknecht S., Gerding H. R., Karreman C., Drescher M., Lashuel H. A., Outeiro T. F., Di Monte D. A., and Leist M. (2013) Oxidative and nitrative α-synuclein modifications and proteostatic stress: implications for disease mechanisms and interventions in synucleinopathies. J. Neurochem. 125, 491–511 23452040 10.1111/jnc.12226

[34] Proukakis C., Dudzik C. G., Brier T., MacKay D. S., Cooper J. M., Millhauser G. L., Houlden H., and Schapira A. H. (2013) A novel aS missense mutation in Parkinson disease. Neurology 80, 1062–1064 23427326 10.1212/WNL.0b013e31828727ba PMC3653201

[35] Porcari R., Proukakis C., Waudby C. A., Bolognesi B., Mangione P. P., Paton J. F., Mullin S., Cabrita L. D., Penco A., Relini A., Verona G., Vendruscolo M., Stoppini M., Tartaglia G. G., Camilloni C., et al (2014) The H50Q mutation induces a 10-fold decrease in the solubility of α-synuclein. J. Biol. Chem. 290, 2395–2404 25505181 10.1074/jbc.M114.610527 PMC4303689

[36] Appel-Cresswell S., Vilarino-Guell C., Encarnacion M., Sherman H., Yu I., Shah B., Weir D., Thompson C., Szu-Tu C., Trinh J., Aasly J. O., Rajput A., Rajput A. H., Jon Stoessl A., and Farrer M. J. (2013) α-Synuclein p.H50Q, a novel pathogenic mutation for Parkinson's disease. Mov. Disord. 28, 811–813 23457019 10.1002/mds.25421

[37] Murphy R. C., and Axelsen P. H. (2011) Mass spectrometric analysis of long-chain lipids. Mass Spectrom. Rev. 30, 579–599 21656842 10.1002/mas.20284 PMC3117083

[38] Xiang W., Menges S., Schlachetzki J. C., Meixner H., Hoffmann A. C., Schlötzer-Schrehardt U., Becker C.-M., Winkler J., and Klucken J. (2015) Posttranslational modification and mutation of histidine 50 trigger α-synuclein aggregation and toxicity. Mol. Neurodegener. 10, 8–24 25886189 10.1186/s13024-015-0004-0 PMC4365527

[39] Chavarría C., and Souza J. M. (2013) Oxidation and nitration of α-synuclein and their implications in neurodegenerative diseases. Arch. Biochem. Biophys. 533, 25–32 23454347 10.1016/j.abb.2013.02.009

[40] Subczynski W. K., and Hyde J. S. (1983) Concentration of oxygen in lipid bilayers using a spin-label method. Biophys. J. 41, 283–286 6301572 10.1016/S0006-3495(83)84439-7 PMC1329181

[41] Esterbauer H., Schaur R. J., and Zollner H. (1991) Chemistry and biochemistry of 4-hydroxynonenal, malonaldehyde and related aldehydes. Free Radic. Biol. Med. 11, 81–128 1937131 10.1016/0891-5849(91)90192-6

[42] Uchida K. (2003) Histidine and lysine as targets of oxidative modification. Amino Acids 25, 249–257 14661088 10.1007/s00726-003-0015-y

[43] Trostchansky A., Lind S., Hodara R., Oe T., Blair I. A., Ischiropoulos H., Rubbo H., and Souza J. M. (2006) Interaction with phospholipids modulates α-synuclein nitration and lipid-protein adduct formation. Biochem. J. 393, 343–349 16146428 10.1042/BJ20051277 PMC1383693

[44] Zhu M., Qin Z. J., Hu D., Munishkina L. A., and Fink A. L. (2006) α-Synuclein can function as an antioxidant preventing oxidation of unsaturated lipid in vesicles. Biochemistry 45, 8135–8142 16800638 10.1021/bi052584t

[45] Liu F., Hindupur J., Nguyen J. L., Ruf K. J., Zhu J., Schieler J. L., Bonham C. C., Wood K. V., Davisson V. J., and Rochet J. C. (2008) Methionine sulfoxide reductase A protects dopaminergic cells from Parkinson's disease-related insults. Free Radic. Biol. Med. 45, 242–255 18456002 10.1016/j.freeradbiomed.2008.03.022 PMC2518045

[46] Khalaf O., Fauvet B., Oueslati A., Dikiy I., Mahul-Mellier A. L., Ruggeri F. S., Mbefo M. K., Vercruysse F., Dietler G., Lee S. J., Eliezer D., and Lashuel H. A. (2014) The H50Q mutation enhances α-synuclein aggregation, secretion, and toxicity. J. Biol. Chem. 289, 21856–21876 24936070 10.1074/jbc.M114.553297 PMC4139205

[47] Vilar M., Chou H. T., Lührs T., Maji S. K., Riek-Loher D., Verel R., Manning G., Stahlberg H., and Riek R. (2008) The fold of α-synuclein fibrils. Proc. Natl. Acad. Sci. U.S.A. 105, 8637–8642 18550842 10.1073/pnas.0712179105 PMC2438424

[48] Comellas G., Lemkau L. R., Nieuwkoop A. J., Kloepper K. D., Ladror D. T., Ebisu R., Woods W. S., Lipton A. S., George J. M., and Rienstra C. M. (2011) Structured regions of α-synuclein fibrils include the early-onset Parkinson's disease mutation sites. J. Mol. Biol. 411, 881–895 21718702 10.1016/j.jmb.2011.06.026 PMC3157309

[49] Zarbiv Y., Simhi-Haham D., Israeli E., Elhadi S. A., Grigoletto J., and Sharon R. (2014) Lysine residues at the first and second KTKEGV repeats mediate α-Synuclein binding to membrane phospholipids. Neurobiol. Dis. 70, 90–98 24905915 10.1016/j.nbd.2014.05.031

[50] Bartels T., Ahlstrom L. S., Leftin A., Kamp F., Haass C., Brown M. F., and Beyer K. (2010) The N-terminus of the intrinsically disordered protein α-synuclein triggers membrane binding and helix folding. Biophys. J. 99, 2116–2124 20923645 10.1016/j.bpj.2010.06.035 PMC3042581

[51] Fusco G., De Simone A., Gopinath T., Vostrikov V., Vendruscolo M., Dobson C. M., and Veglia G. (2014) Direct observation of the three regions in α-synuclein that determine its membrane-bound behaviour. Nat. Commun. 5, 3827 24871041 10.1038/ncomms4827 PMC4046108

[52] Stefanis L. (2012) α-Synuclein in Parkinson's disease. Cold Spring Harb. Perspect. Med. 2, a009399 22355802 10.1101/cshperspect.a009399 PMC3281589

[53] De Franceschi G., and Polverino de Laureto P. (2014) Role of different regions of α-synuclein in the interaction with the brain fatty acid DHA. J. Chromatograph. Separat. Techniq. 5, 219–226

[54] Miyashita K. (2014) Paradox of omega-3 PUFA oxidation. Eur. J. Lipid Sci. Technol. 116, 1268–1279

[55] Sharon R., Bar-Joseph I., Mirick G. E., Serhan C. N., and Selkoe D. J. (2003) Altered fatty acid composition of dopaminergic neurons expressing α-synuclein and human brains with α-synucleinopathies. J. Biol. Chem. 278, 49874–49881 14507911 10.1074/jbc.M309127200

[56] Assayag K., Yakunin E., Loeb V., Selkoe D. J., and Sharon R. (2007) Polyunsaturated fatty acids induce α-synuclein-related pathogenic changes in neuronal cells. Am. J. Pathol. 171, 2000–2011 18055555 10.2353/ajpath.2007.070373 PMC2111122

[57] Leong S. L., Pham C. L., Galatis D., Fodero-Tavoletti M. T., Perez K., Hill A. F., Masters C. L., Ali F. E., Barnham K. J., and Cappai R. (2009) Formation of dopamine-mediated α-synuclein-soluble oligomers requires methionine oxidation. Free Radic. Biol. Med. 46, 1328–1337 19248830 10.1016/j.freeradbiomed.2009.02.009

[58] Binolfi A., Limatola A., Verzini S., Kosten J., Theillet F.-X., Rose H. M., Bekei B., Stuiver M., van Rossum M., and Selenko P. (2016) Intracellular repair of oxidation-damaged α-synuclein fails to target C-terminal modification sites. Nat. Commun. 7, 10251 26807843 10.1038/ncomms10251 PMC4737712

[59] Lukiw W. J., and Bazan N. G. (2008) Docosahexaenoic acid and the aging brain. J. Nutr. 138, 2510–2514 19022980 10.3945/jn.108.096016 PMC2666388

[60] Darios F., Ruipérez V., López I., Villanueva J., Gutierrez L. M., and Davletov B. (2010) α-Synuclein sequesters arachidonic acid to modulate SNARE-mediated exocytosis. EMBO Rep. 11, 528–533 20489724 10.1038/embor.2010.66 PMC2897113

[61] Gill S. C., and von Hippel P. H. (1989) Calculation of protein extinction coefficients from amino acids sequence data. Anal. Biochem. 182, 319–326 2610349 10.1016/0003-2697(89)90602-7

[62] Uversky V. N., Yamin G., Souillac P. O., Goers J., Glaser C. B., and Fink A. L. (2002) Methionine oxidation inhibits fibrillation of human α-synuclein *in vitro*. FEBS Lett. 517, 239–244 12062445 10.1016/s0014-5793(02)02638-8

[63] Means G. E., and Feeney R. E. (1971) Chemical Modification of Proteins, Vol. 2, Holden-Day Inc., San Francisco

